# Advances in cholera research: from molecular biology to public health initiatives

**DOI:** 10.3389/fmicb.2023.1178538

**Published:** 2023-05-22

**Authors:** Madison G. Walton, Isabella Cubillejo, Dhrubajyoti Nag, Jeffrey H. Withey

**Affiliations:** Department of Biochemistry, Microbiology, and Immunology, Wayne State University School of Medicine, Detroit, MI, United States

**Keywords:** cholera, *Vibrio cholerae*, animal models, bacterial pathogenesis, aquatic bacteria

## Abstract

The aquatic bacterium *Vibrio cholerae* is the etiological agent of the diarrheal disease cholera, which has plagued the world for centuries. This pathogen has been the subject of studies in a vast array of fields, from molecular biology to animal models for virulence activity to epidemiological disease transmission modeling. *V. cholerae* genetics and the activity of virulence genes determine the pathogenic potential of different strains, as well as provide a model for genomic evolution in the natural environment. While animal models for *V. cholerae* infection have been used for decades, recent advances in this area provide a well-rounded picture of nearly all aspects of *V. cholerae* interaction with both mammalian and non-mammalian hosts, encompassing colonization dynamics, pathogenesis, immunological responses, and transmission to naïve populations. Microbiome studies have become increasingly common as access and affordability of sequencing has improved, and these studies have revealed key factors in *V. cholerae* communication and competition with members of the gut microbiota. Despite a wealth of knowledge surrounding *V. cholerae*, the pathogen remains endemic in numerous countries and causes sporadic outbreaks elsewhere. Public health initiatives aim to prevent cholera outbreaks and provide prompt, effective relief in cases where prevention is not feasible. In this review, we describe recent advancements in cholera research in these areas to provide a more complete illustration of *V. cholerae* evolution as a microbe and significant global health threat, as well as how researchers are working to improve understanding and minimize impact of this pathogen on vulnerable populations.

## Graphical Abstract

Overview of the *Vibrio cholerae* life cycle and the topics covered in this review. Figure prepared using BioRender.

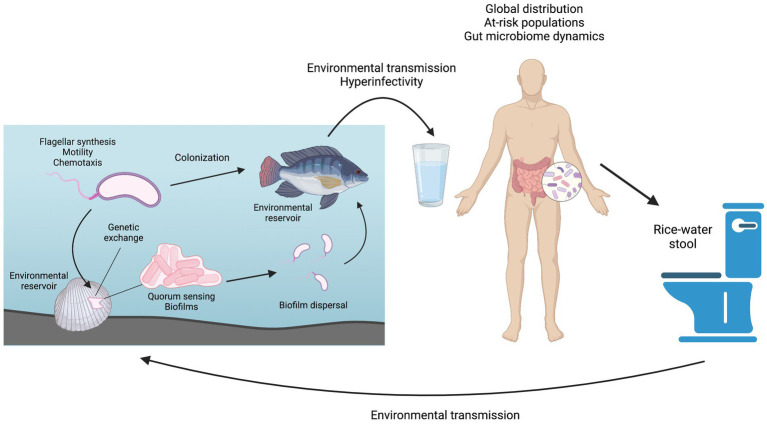

## Introduction

Cholera is an acute diarrheal disease with 2.9 million cases and 95,000 deaths estimated to occur each year in at least 47 countries across the world (Ali et al., 2015). The majority of severe cases occur in children under 5 years old. Cholera is often described as a disease of inequity, disproportionately affecting the poorest populations of a country or community (GTFCC, 2017). This longstanding disease has been thoroughly studied in a wide range of research fields from basic science to therapeutics. Mechanisms for prevention, intervention, and possible elimination of cholera have been clearly described and continue to be investigated but lack practical implementation in many vulnerable populations. The etiological agent of cholera is the gram-negative bacterium *Vibrio cholerae*, specifically those strains belonging to serogroups O1 and O139. Strains belonging to other serogroups may cause less severe non-cholera diarrhea or no disease symptoms at all and are collectively referred to as non-O1/non-O139 strains. *V. cholerae* is highly motile in aquatic environments, using a single, polar flagellum to propel itself (Echazarreta and Klose, 2019). This bacterium is also readily found in biofilms that form on hard surfaces, i.e., rocks or pipes, as well as in association with shellfish and vertebrate fish (Baine et al., 1974; Silva and Benitez, 2016). Humans can become infected with *V. cholerae* O1/O139 by consuming contaminated food or water, granting the bacteria access to the small intestine. Here, the bacteria aggregate using the toxin co-regulated pilus (TCP) and other colonization factors to colonize the intestine in a non-invasive manner (Almagro-Moreno et al., 2015; Silva and Benitez, 2016). Once established in the small intestine, the pathogen induces production of cholera toxin (CT), which results in an ion imbalance in the host intestine, leading to the rapid loss of fluids and electrolytes and potentially deadly dehydration *via* profuse, watery diarrhea (Thiagarajah and Verkman, 2005). Bacteria are disseminated from the intestine with the diarrhea, known as “rice water stool,” of an infected patient and exhibit hypervirulence for a limited time period (Butler et al., 2006). In many cases, cholera-containing fecal matter then contaminates a shared drinking water source, enabling infection with *V. cholerae* O1/O139 to spread rapidly through an entire community in the form of localized outbreaks (GTFCC, 2017). This massive fluid loss can lead to hypovolemia and can be up to 50% lethal if untreated (Sack et al., 2004). Fortunately, fatality rates drop significantly, to just 1%–2%, with standard treatment using an oral rehydration solution (ORS) (Desjeux et al., 1997). Prompt implementation of ORS therapy counteracts the fluid and electrolyte loss caused by *V. cholerae* infection and keeps the patient alive while the body naturally clears the infection over a period of several days.

Over the past 200 years, seven pandemics of cholera have been recorded, though instances of cholera-like illness have been described for millennia (Blake, 1994). The long-established nature of this disease has enabled evolutionary differentiation of the pathogen into thousands of strains ranging from environmental strains to those capable of causing endemic and pandemic cholera.

Cholera is estimated to cost $2 billion each year in global healthcare costs and loss of productivity (GTFCC, 2017). As global society becomes increasingly interconnected, cholera is perpetuated by human travel and transmission to naïve populations, lack of adequate infrastructure or disruption of existing infrastructure due to poverty, war, or natural disaster, and shifting weather patterns resulting from climate change. Fortunately, an abundance of research continues to emerge to better describe this pathogen and the disease it causes. This review aims to describe recent advancements in cornerstone cholera research related to genetic evolution of *V. cholerae* strains, molecular mechanisms of pathogenesis, improvements in animal models, pathogen interactions with the host microbiome and immune response, and disease epidemiology, presentation, and mitigation initiatives.

## *Vibrio cholerae* evolutionary genomics

The *Vibrio cholerae* genome is approximately 4.0 MB in size and organized into two, distinct circular chromosomes (Trucksis et al., 1998; Heidelberg et al., 2000). Nearly 75% of the genome is contained on chromosome 1 with the remainder on chromosome 2, though a few isolates have been identified that contain a single, fused chromosome and are termed Natural Single Chromosome Vibrio (Chapman et al., 2015; Johnson et al., 2015; Yamamoto et al., 2018; Sozhamannan and Waldminghaus, 2020). While strain-specific differences exist, the entire *V. cholerae* genome generally contains between 3,600 and 3,900 coding sequences (Heidelberg et al., 2000; Thompson et al., 2011). Chromosome 1 serves as the core genome, encoding most of the essential housekeeping genes and conserved virulence genes. In contrast, chromosome 2 encodes many hypothetical proteins and open reading frames that appear to have been obtained from external sources through horizontal gene transfer events (Heidelberg et al., 2000). While many chromosome 2 genes serve redundant or unknown functions, at least a dozen essential genes have been identified on this chromosome and encode ribosomal proteins L35 and L20 as well as an NAD synthetase and ParA family protein involved in chromosome partitioning (Cameron et al., 2008; Hui et al., 2010; Chao et al., 2013; Kamp et al., 2013). Strains of *V. cholerae* are classified into serogroups according to the unique structure of the O-antigen associated with the strain’s outer membrane lipopolysaccharide (LPS) molecule. In a recent study, Murase et al. (2022) explored the genetic relatedness of all 210 reported serogroups and identified critical distinctions in structural biosynthesis gene clusters on both chromosomes. While only the O1 and O139 serogroups have been known to cause pandemic cholera, members of the remaining serogroups have had significant impacts as environmental strains. While not typically life-threatening, non-O1/non-O139 serogroups can cause sporadic cases of non-cholera diarrhea, sometimes closely resembling cholera, and some have been shown to act as evolutionary intermediaries in virulence gene acquisition *via* homologous recombination and horizontal gene transfer (Li et al., 2014, 2019a).

In the environment, *V. cholerae* is often associated with chitinous surfaces, such as those found on mollusks and other shellfish, and with phytoplankton (Baine et al., 1974; Tamplin et al., 1990; Hounmanou et al., 2019). Vertebrate fish have more recently been proposed as potential *V. cholerae* reservoirs, as both environmental and toxigenic strains have been isolated from numerous fish species, including tilapia (*Oreochromis niloticus*) (Senderovich et al., 2010; Halpern and Izhaki, 2017). Zebrafish (*Danio rerio*) have been shown to be naturally susceptible to infection and colonization by *V. cholerae* and have been developed as a *V. cholerae* model (Runft et al., 2014; Mitchell et al., 2017; Mitchell and Withey, 2018; Nag et al., 2018b, 2020). Aquatic reservoirs harboring both environmental and pandemic *V. cholerae* strains provide rich conditions for genetic evolution, and many non-O1/non-O139 have been found to contain partial pathogenicity islands and fully intact virulence genes, including the Vibrio seventh pandemic islands 1 and 2 (VSP-1, VSP-2), toxin co-regulated pilin (*tcpA*), hemolysin A (*hlyA*), and the Type 6 secretion system (T6SS) (Li et al., 2014, 2019a; Yan et al., 2022; Santoriello et al., 2023).

A mixed-transmission dynamic model of *V. cholerae* developed by Mavian et al. (2020) made use of spatiotemporal *V. cholerae* distribution following the single-source introduction in Haiti to model the establishment of aquatic reservoirs and the potential for evolutionary gene transfer events. These reservoirs are particularly relevant in cholera-endemic countries where seasonal cholera blooms result in multiple, periodic outbreaks with strains of varying virulence. Ongoing susceptibility to infection despite previous exposure to *V. cholerae* has been associated with serotype switching—a phenomenon in which *V. cholerae* O1 serogroup strains can express alternative surface antigens to present as either Ogawa, Inaba, or very rarely Hikojima serotypes—and is likely enabled by gene transfer under selective conditions in environmental reservoirs during off-peak cholera seasons (Baddam et al., 2020; Ramamurthy et al., 2020). Non-O1/non-O139 strains that primarily exist in the environment may use the same virulence genes in a different manner. For example, the recently identified T6SS gene cluster known as Aux3 has been shown to readily excise from the genome and recombine in a new location. However, this activity is typically only observed in environmental strains, while pandemic strains have integrated the feature into the chromosome (Santoriello et al., 2020).

From 1817 until the 1960s, pandemic cholera was caused by the *V. cholerae* O1 Classical biotype, characterized by the presence of a distinctive CT, TCP, and Vibrio pathogenicity island (VPI). Cholera in the current, ongoing seventh pandemic has been caused by a new O1 biotype, El Tor. El Tor biotype emerged as the primary causative agent of pandemic cholera beginning in 1961 and is defined by its resistance to polymyxin B, production of hemolysin A, and presence of two unique pathogenicity islands, VSP-1 and VSP-2, all of which Classical biotype lacks (Chart, 2012). Several diagnostic methods have been developed to distinguish between Classical and El Tor biotypes in recent years. PCR-based genotypic assays typically screen for specific sequence variations in virulence genes including *tcpA*, *ctxA*, *ctxB*, and *toxR*, while another genome-based method targets unique small RNA genes (Crumfield et al., 2018; Greig et al., 2018; Ahmed et al., 2019). Biotype can be distinguished phenotypically by evaluating antibiotic and phage susceptibility, capability for hemolysis and proteolysis, and variations in metabolism of citrate and glucose (Crumfield et al., 2018; Lee et al., 2020). One simple diagnostic measure for distinguishing between Classical and El Tor biotype strains in clinical settings has been the susceptibility of Classical strains to polymyxin B while El Tor strains have demonstrated resistance to this antibiotic, though this may not always be a reliable metric as El Tor strains continue to evolve (Crumfield et al., 2018).

When *V. cholerae* was first introduced to Haiti, it had devastating effects on the population which, initially, was largely attributed to the naivety of the previously unexposed region. While this certainly played a role in the rapid transmission and severe disease observed during this outbreak, Haitian variants of El Tor have been identified as more virulent than their southeastern Asian predecessors inducing elevated levels of inflammation and damage to the intestinal mucosa (Ghosh et al., 2019). These variants have also demonstrated greater production of CT, increased motility, and enhanced colonization dynamics in both human disease and animal host models (Satchell et al., 2016; Ghosh et al., 2019). Interestingly, recent epidemics in India and West Bengal have been caused by El Tor strains that more closely resemble Haitian variant strains in their genetic profile but also exhibit polymyxin B-sensitivity (Samanta et al., 2018; Shaw et al., 2022). These polymyxin B-sensitive strains have also exhibited hypervirulent traits compared to El Tor strains previously isolated in the same regions of southeast Asia (Samanta et al., 2018). These Classical-like features of more recently evolved El Tor isolates will likely require the development of new measures for phenotypic identification of biotype, particularly in resource-limited settings. Additionally, five clinical isolates from Kolkata, India revealed the inability to replicate the cholera toxin phage (CTXΦ) for the secretion of infectious particles by some El Tor variants, a feature that was commonly observed in El Tor strains between the 1970s and early 2010s (Ochi et al., 2021).

With the rapid development and decreasing cost of genome sequencing and analysis in the early 2000s, extensive genomic comparison analyses have described the evolution of the El Tor biotype and its evolutionary successors in detail. Whole-genome studies have revealed the similarities and differences in key virulence factors, including the CTXΦ, and have used these data to characterize the evolution and spread of El Tor variants into three waves (Kim et al., 2014). Hu et al. (2016) detailed key genomic events between the early 1900s and the 1960s that enabled El Tor’s maturation from a relatively benign form of *V. cholerae* to the virulent pathogen credited with causing the ongoing 7th pandemic of cholera. These events included the acquisition of an El Tor-specific *tcpA* gene that enabled human colonization, pathogenicity islands VSP-1 and VSP-2, and an El Tor-specific CTXΦ. Since this initial characterization of the lineage leading to the 7th pandemic by El Tor, others have explored genetic variation within El Tor strains to assess virulence trait acquisition. Large genomic fragments carrying genes for antimicrobial resistance, including the integrative and conjugative element (ICE) known as the SXT element, have been acquired by some El Tor strains isolated from the natural environment, and afford resistance to tetracycline, streptomycin, and even chloramphenicol (Ahmed et al., 2005; Sarkar et al., 2019).

An analysis of over 300 *V. cholerae* O1 strains revealed El Tor strains typically contained more virulence-related genes than Classical strains, as well as the presence of many redundant genes across the El Tor genome (Li et al., 2019b). While *V. cholerae* readily takes up DNA from the environment, defense mechanisms have also been developed to prevent the acquisition of unwanted or detrimental features. Both small, multicopy- and large, low copy number plasmids can be degraded by defense mechanisms identified as DdmABC and DdmDE should they prove to be detrimental to overall fitness of the *V. cholerae* host cell (Jaskólska et al., 2022). Phage defense in El Tor strains has been attributed in part to activity of genes on the VSP-1 and VSP-2 pathogenicity islands, including an antiviral cytidine deaminase which disrupts normal availability of nucleotides to deprive infecting phage of necessary components to replicate (Hsueh et al., 2022; O’Hara et al., 2022).

The natural habitat of *V. cholerae* in biofilms is often composed of richly diverse communities of *Vibrio* species and other aquatic bacteria. Some of the species present in these biofilms are capable of natural competence and can release significant amounts of DNA (>100 μg/ml) into the environment. Biofilms readily form on chitinous surfaces, a biopolymer that has been shown to induce natural competence in some *V. cholerae* strains (Baur et al., 1996; Meibom et al., 2005). Additionally, *V. cholerae* killing of non-kin bacterial competitors mediated by the type six secretion system (T6SS) enables the uptake of large DNA fragments (>150 Kbp) following lysis of the target cell (Joshi et al., 2017; Matthey et al., 2019). Exposure to high concentrations of free DNA in these chitin-rich conditions enables the rapid genomic diversification of *V. cholerae* which is modeled in the evolution of El Tor strains.

## Control of *Vibrio cholerae* virulence by human gut signals

In the aquatic environment, *V. cholerae* does not produce the human-specific virulence factors that are required to cause cholera. After ingestion by a human, the bacteria sense signals in the gut that initiate a complex cascade of transcription factors that ultimately induce production of the major virulence factors, CT and TCP, together with a collection of other accessory virulence factors (Matson et al., 2007). At the top of the cascade are transcription factors AphA and AphB (Kovacikova and Skorupski, 1999; Skorupski and Taylor, 1999; Kovacikova and Skorupski, 2001; Kovacikova et al., 2004). AphA is translated at low cell density, and its genomic targets were recently described using CHiP-Seq (Haycocks et al., 2019). AphB senses low oxygen and low pH and becomes active as *V. cholerae* passes through the stomach and into the upper small intestine (Kovacikova et al., 2010). AphA and AphB work together to activate transcription of the next level of the cascade, which is composed of the TcpPH and ToxRS pairs of integral membrane proteins. While ToxRS is thought to be constitutively produced, TcpPH production requires the activity of AphA and AphB. When produced, TcpPH senses the bile salt taurocholate (Yang et al., 2013; Xue et al., 2016), whereas ToxRS senses other bile salts in the intestinal lumen (Midgett et al., 2017; Bina et al., 2021). TcpP and ToxR then bind directly to the promoter of the master virulence regulator, ToxT, and activate its production (Krukonis et al., 2000; Krukonis and DiRita, 2003; Goss et al., 2010; Fan et al., 2014; Haas et al., 2015). ToxT binds directly to the promoters upstream of *ctxAB* and *tcpA* and activates their transcription. However, ToxT activity is repressed by unsaturated fatty acid components of bile to prevent virulence factor production in the lumen of the small intestine (Chatterjee et al., 2007; Koestler and Waters, 2014; Plecha and Withey, 2015). As motile *V. cholerae* enters the mucus layer, the large fatty acids cannot penetrate, and ToxT becomes activated by the presence of bicarbonate, which is secreted by epithelial cells (Abuaita and Withey, 2009). Unsaturated fatty acids and bicarbonate have opposing roles in affecting the affinity of ToxT for its DNA binding sites (Withey and DiRita, 2006; Thomson and Withey, 2014; Plecha and Withey, 2015; Thomson et al., 2015). Thus, CT and TCP production only occurs when *V. cholerae* has reached the ideal location for colonization, within the intestinal mucus layer and close to the epithelial surface in crypts, where CT can enter cells and play its toxic role, resulting in voluminous watery diarrhea (Millet et al., 2014).

## Advances in cellular and molecular biology of the *Vibrio cholerae* life cycle

*Vibrio cholerae* has two distinct phases in its lifecycle: the highly motile, free-swimming state, and the sessile, virulent state. Motility is important in an aqueous environment, while attachment and biofilm formation is necessary for colonization in the human small intestine or on the surfaces of fish, plankton, and other chitinous material (Tamplin et al., 1990; Donlan and Costerton, 2002; Senderovich et al., 2010; Hathroubi et al., 2017). Responding to environmental signaling is crucial for *V. cholerae* survival, and robust methods of inverse regulation over virulence factors and motility are required.

### Flagellar synthesis, motility, and chemotaxis

In an aqueous environment, *V. cholerae* is highly motile due to a single, sheathed, polar flagellum. The flagellum is powered by a protein complex in the membrane, called the motor, that uses the transmembrane sodium motive force to generate torque (Terashima et al., 2008; Halang et al., 2013). This causes rotational movements that can propel *V. cholerae* up to 60 cell-body lengths per second (McCarter, 2001). Other movements include twitching motility, which is dependent on pili, and gliding motility, which is independent of pili or flagella (Mattick, 2002; Nan and Zusman, 2011). This can be utilized by *V. cholerae* for moving in different media or for adherence (Butler and Camilli, 2005). The *V. cholerae* flagellum is being explored for its role in colonization and pathogenicity. Expression of major *V. cholerae* virulence factors have long been known to be inversely regulated with expression of flagellar genes, and *V. cholerae* that are actively colonizing intestinal epithelium typically do not have flagella.

The *V. cholerae* flagellum has recently been shown to secrete MakA, a motility-associated toxin. From the same gene cluster, proteins MakA, MakB, and MakE can form a tripartite cytolytic toxin *in vitro*, *via* membrane binding and assembly of a pore (Nadeem et al., 2021). The activation or presence of a flagellum can also influence biofilm development in an inverse manner. *V. cholerae* mutants that lacked a functional flagellum formed colonies with a morphological switch to a rugose colony, which is associated with expression of extracellular polysaccharides similar to biofilms (Echazarreta and Klose, 2019).

Movement of the *V. cholerae* flagellum is another area of study. The flagellum is perpetually rotating, and sodium concentration of the environment directly affects swimming speed (Halang et al., 2013; Grognot et al., 2021). *V. cholerae* is able to perform different swim patterns that result in either a more random dispersal or a more targeted movement near surfaces. This may be an advantage, perhaps in the event of chemotaxis, outcompeting other bacteria, or to find a viable surface for attachment (Utada et al., 2014; Grognot et al., 2021). *V. cholerae* has most chemotaxis genes organized into 3 operons, which allow motile *V. cholerae* to adjust its direction according to environmental signals (Heidelberg et al., 2000; Butler and Camilli, 2004) Similarly, non-chemotactic *V. cholerae* El Tor biotype mutants outcompeted wild type strains in the infant mouse small intestine, indicating that chemotaxis significantly inhibits colonization overall. However, colonization was localized aberrantly (Butler and Camilli, 2005). In particular, smooth swimming is crucial to competition in the small intestine of infant mice (Butler and Camilli, 2004). Chemotaxis is also relevant to differentiate among surfaces. Valiente et al. identified an accessory colonization gene in *V. cholerae* O1 El Tor, *acfC*, that encodes a methyl-accepting chemotaxis protein. *In vitro*, AcfC induced chemotaxis towards intestinal mucin but not chitin (Valiente et al., 2018). In the environment, *V. cholerae* uses the flagellum to swim to chitinous surfaces, and attaches irreversibly with the flagellum and mannose-sensitive hemagglutinin (MSHA) type IV pili (Utada et al., 2014). The second messenger cyclic di-GMP (c-di-GMP) is crucial to how *V. cholerae* responds to the environment, affecting the concentration and binding of transcriptional regulators (Conner et al., 2017; Homma and Kojima, 2022). CsrA is an RNA-binding protein that regulates c-di-GMP metabolism, which inversely regulates flagellar gene expression in *V. cholerae*, and directly regulates virulence gene expression (Tischler and Camilli, 2005; Jonas et al., 2008; Butz et al., 2021). Low levels of c-di-GMP promote FlrA, which is required for flagellar gene expression and motility (Yildiz and Visick, 2009; Conner et al., 2017). High levels of c-di-GMP repress motility and virulence, and activate biofilm matrix production, as CsrA controls polysaccharide production depending on the *V. cholerae* growth phase (Hunter et al., 2014; Conner et al., 2017; Echazarreta and Klose, 2019).

### Quorum sensing and regulation

Once attached to a biotic or an abiotic surface, *V. cholerae* switches from a free-swimming planktonic bacterium to form aggregates. This transition initiates colonization. Depending on cell density, bacteria can communicate in a cell-to-cell manner to coordinate responses to the environment *via* a process called quorum sensing (Papenfort and Bassler, 2016). This relies on the secretion and detection of diffusible signaling molecules called autoinducers.

In *V. cholerae*, there are four histidine kinases that act as redundant quorum sensing receptors, LuxPQ, CqsS, CqsR and VpsS (Watve et al., 2020). Four synthases, LuxM, LuxS, Cqs, and Tdh, produce autoinducers AI-1, AI-2, CAI-1, and DPO (Mukherjee and Bassler, 2019). At a low cell density, autoinducer concentration is low, and virulence genes are expressed (Watve et al., 2020). A phosphorylation cascade activates production of AphA, a transcription factor at the top of the complex virulence regulatory cascade (Haycocks et al., 2019; Mukherjee and Bassler, 2019; Mashruwala and Bassler, 2020). At high cell density, the autoinducer concentration is high, which promotes cell-to-cell coordination (Mashruwala and Bassler, 2020). In *V. cholerae*, the quorum sensing regulator HapR is produced to repress AphA synthesis and reduce virulence gene expression, as has been demonstrated in *Drosophila* and other models (Rutherford et al., 2011). HapR also represses expression of Vibrio polysaccharide (VPS) matrix enzymes, as demonstrated in a *Drosophila* host (Zhu et al., 2002; Vance et al., 2003; Kamareddine et al., 2018). Judger et al. suggests that quorum sensing activates HapR to repress tryptophan uptake, which cascades to activate a commensal relationship from the *Drosophila* host innate immune system *via* serotonin production (Jugder et al., 2022).

The presence or absence of autoinducers can indicate environmental changes. CAI-1 is used for intra-genus communication, and AI-2 and DPO are used for inter-species communication. In anaerobic conditions, *V. cholerae* produces DPO but not CAI-1, while the opposite is true in aerobic conditions (Mashruwala and Bassler, 2020). This signaling is relevant to a free-swimming lifestyle in water, versus the anaerobic conditions of the human small intestine, for example.

### Biofilm formation and regulation

The presence of mucin or chitin causes *V. cholerae* to grow outward from the founder cell, with the resulting mechanical pressure causing surface adhesion (Valiente et al., 2018; Yan and Bassler, 2019). High cell density induces high levels of c-di-GMP, which repress flagellar genes. VpsR and VpsT then activate to form a surface-associated aggregation called a biofilm (Hunter et al., 2014; Silva and Benitez, 2016; Conner et al., 2017). Biofilm cells are in a three-dimensional matrix made of polysaccharides, proteins, and extracellular DNA. This protective matrix allows for surface adhesion, enzyme proximity for metabolism, and potential horizontal gene transfer (Flemming and Wingender, 2010). *V. cholerae* in biofilm has also been shown to be hyper-infectious when compared to free-swimming *V. cholerae* (Tamayo et al., 2010). Human stool from confirmed cholera cases has both planktonic bacteria and clumped bacteria, indicating that aggregation or biofilm-like behavior may be occurring in the human gut (Faruque et al., 2006; Nelson et al., 2007).

A redundant set of four proteases control the timing of *V. cholerae* to either form a scaffold or use motility to aggregate into the scaffold (Jemielita et al., 2018). This further illuminates how *V. cholerae* persists in the water and how it colonizes the human small intestine. By culturing *V. cholerae* on different surfaces and comparing the subsequent biofilms to free-swimming cells when infecting infant mouse intestine, biofilm cells enhanced expression of virulence factors including the TCP (Gallego-Hernandez et al., 2020). The TCP is a major *V. cholerae* virulence factor required for human colonization (Kaper et al., 1995; Thelin and Taylor, 1996; Teschler et al., 2015).

Biofilm formation can also depend on extracellular components and certain proteases. In one study, extracellular signaling molecule accumulation was prevented *via* a continuously refreshed system. This identified a difference in biofilm gene regulation in *V. cholerae*, depending on dynamic versus static conditions (Seper et al., 2014). Extracellular nucleases were also found to regulate the amount of extracellular DNA involved in both developing and dispersing biofilm (Seper et al., 2011). These nucleases were also necessary for hyper-infectivity from biofilm-formed *V. cholerae*. Kitts et al. identified a calcium-dependent protease, LapG, which cleaves adhesins FrhA and CraA. A *V. cholerae* mutant lacking LapG increased the amount of biofilm formed, indicating the protease mediation in biofilm formation (Kitts et al., 2019).

### Biofilm dispersal

Through mutagenesis, proteins from three classes of genes were identified to play a sequential role in biofilm dispersal: signal-transduction proteins, matrix-degradation enzymes, and motility factors (Bridges and Bassler, 2021). Dispersal is the last step following the sessile, biofilm phase in the *V. cholerae* life cycle. Mechanical changes in the environment such as flow rate can regulate *V. cholerae* biofilm production or dispersal. In nutrient medium with an increased flow rate, biofilm mass increased. If the flow rate was slowed or stopped, *V. cholerae* within biofilms dispersed from the outer surface (Singh et al., 2017).

Access to soluble nutrients also determines dispersal. RpoS protein, an alternate sigma factor, regulates a generalized stress response during starvation and is essential for *V. cholerae* to detach from mucus when it reaches a high cell density (Nielsen et al., 2006; Müller et al., 2007). During the stressor of low nutrient conditions, elevated RpoS was detected (Singh et al., 2017). Species-specific polyamines are also involved in biofilm development and repression (Lee et al., 2009). Periplasmic polyamine sensor MbaA, and polyamine reporter PotD1 regulate *V. cholerae* biofilm dispersal, with PotD1 being essential to activating dispersal (Bridges and Bassler, 2021). Excreted *V. cholerae* from humans, mice, or other animals has shown hyper-infectivity during the transmission to the next host. Detachment and dispersal from the biofilm into the environment are also linked to the hyper-infectivity of *V. cholerae* during transmission to the host from the environment (Alam et al., 2005; Butler et al., 2006; Tamayo et al., 2010).

## Improvements in animal models for studying *Vibrio cholerae* infection, colonization, disease, and transmission

Animal models for cholera research can be divided into two categories: mammalian models and non-mammalian models. These models, their uses, and their advantages and disadvantages are summarized in Table 1. The most commonly used mammalian model for enteric diseases generally is the specific-pathogen free (SPF) adult mouse (*Mus musculus*), but these hosts can be resistant to intestinal colonization by some strains of orally inoculated *V. cholerae*, due in part to the presence of gut microbiota (Sit et al., 2019). The streptomycin-treated adult mouse is sometimes used as a model for *V. cholerae* colonization. However, in this model *V. cholerae* colonizes the colon but not the small intestine (SI), where *V. cholerae* colonizes during human infection. This model is TCP-independent, and there are no detectable disease symptoms (Nygren et al., 2009; Wang et al., 2018). Pretreatment with clindamycin or ketamine to disrupt intestinal microbiota and motility can permit oral *V. cholerae* infection in the adult mouse (Olivier et al., 2009; You et al., 2019). Clindamycin is used as an antibiotic, like streptomycin, to clear the gut microbiota, and ketamine is a dissociative anesthetic that can be used to slow down the bowel movement and give *V. cholerae* a better chance to colonize. The most widely used mammalian model for *V. cholerae* colonization is the 3- to 5-day-old infant mouse, where oral challenge of *V. cholerae* can initiate TCP-dependent colonization of the SI (not the colon) and CT-dependent fluid accumulation within 16 h (Angelichio et al., 1999; Ritchie and Waldor, 2009). Advanced microscopy of the infant mouse small intestinal tissue has revealed the localization patterns of *V. cholerae* in the intestine control virulence gene regulation during infection (Millet et al., 2014; Gallego-Hernandez et al., 2020). Different competition studies in the infant mouse model have recently emphasized the contributions of fatty acid and carbon metabolism, cell wall maintenance, and *V. cholerae* LPS modifications during intestinal colonization (Hayes et al., 2017; Wang et al., 2018; Fleurie et al., 2019; Yang et al., 2020; Sit et al., 2021).

**Table 1:** Summary of animal models for *V. cholerae*.

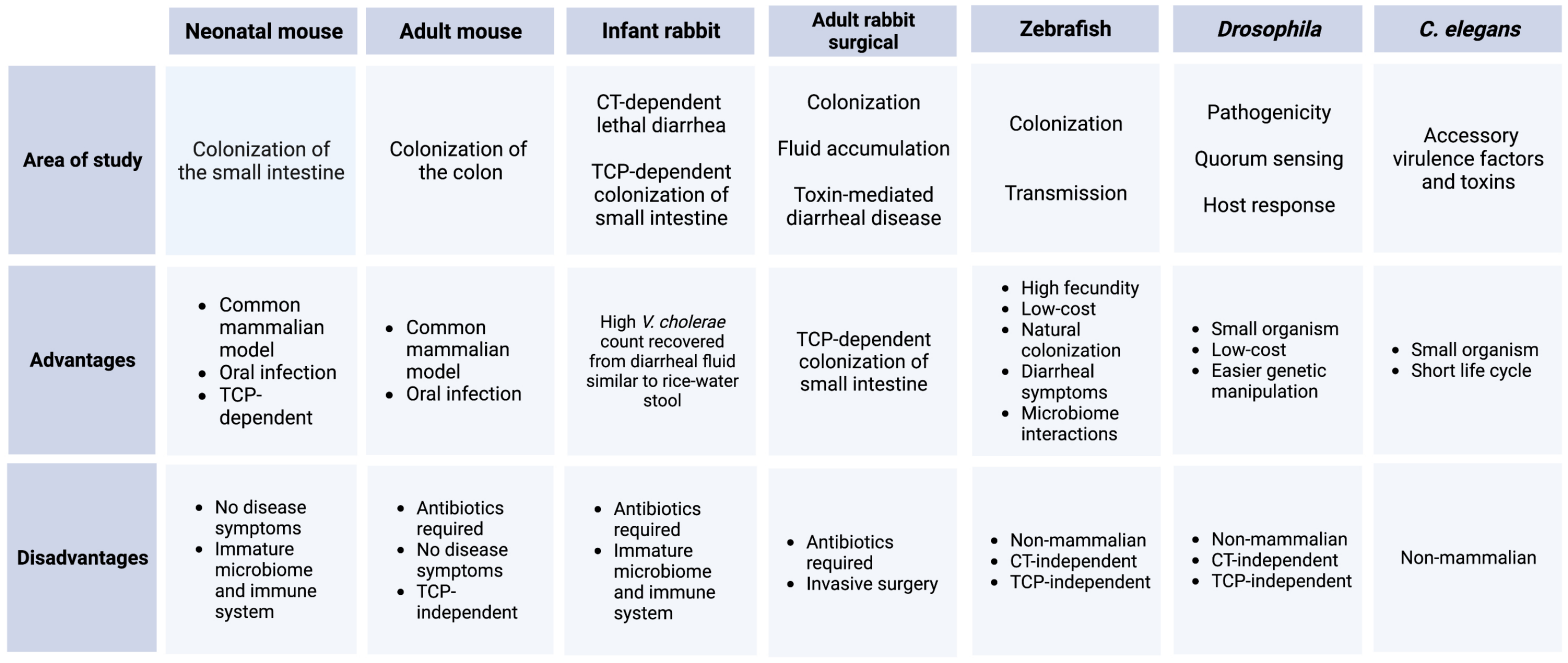

Two adult rabbit surgical models have been widely used for decades to test the colonization and fluid secretion resulting from *V. cholerae* infection. The removable intestinal tie adult rabbit diarrhea (RITARD) model is used for studying the toxin-mediated diarrheal disease caused by *V. cholerae* (Spira et al., 1981; Sinha et al., 2015). The ileal loop model is a widely used surgical model to study the fluid accumulation following by *V. cholerae* colonization by surgically creating sealed loops in the small intestine of the adult rabbit (Burrows and Musteikis, 1966; Mondal et al., 2014; Withey et al., 2015). These adult rabbit surgical models have largely been replaced by the infant rabbit (*Oryctolagus cuniculus*) model of cholera, which exhibits rapid CT-dependent lethal diarrheal illness, along with TCP-dependent SI colonization (Ritchie et al., 2010). High numbers of *V. cholerae* can be readily collected from the infant rabbit diarrheal fluid, which is quite similar to human rice water stool in chemical composition (Ritchie et al., 2010). Infant rabbit studies have revealed the genetic landscape of colonization factors in *V. cholerae* through transposon-insertion sequencing (Tn-Seq) screens; however, these screens can be largely limited by bottleneck effects in infant mice (Fu et al., 2013; Kamp et al., 2013; Pritchard et al., 2014; Hubbard et al., 2018). *V. cholerae* RNA-seq, metabolomic, and proteomic datasets, as well as insights into *V. cholerae* population dynamics during infection, have been successfully studied in the infant rabbit model (Mandlik et al., 2011; Abel et al., 2015; Zoued et al., 2021). Recently, a transcriptomic study in infant rabbits revealed novel roles for CT in the shaping of the pathogen’s nutritional microenvironment (Rivera-Chavez and Mekalanos, 2019).

There are several non-mammalian species for studying *V. cholerae* as well, such as fruit flies (*Drosophila melanogaster*), nematodes (*Caenorhabditis elegans*), wax moths (*Galleria mellonella*), and zebrafish (*Danio rerio*). The intestinal anatomy and immune system of *Drosophila* is similar to mammals, enabling the use of flies to investigate pathogenicity, quorum sensing, and host response to *V. cholerae* infection (Hang et al., 2014; Vanhove et al., 2017; Kamareddine et al., 2018; Davoodi and Foley, 2019). The introduction of CT can induce death in flies although the infection and host-killing by *V. cholerae* in these models is neither CT-dependent nor TCP-dependent (Blow et al., 2005). Even with these limitations, this model is useful as it offers reproducibility, low cost, and easy genetic manipulation compared to mammalian hosts. Cholera research in *C. elegans* is important for studying the known and presumed accessory *V. cholerae* virulence factors and toxins (Vaitkevicius et al., 2006; Logan et al., 2018). The ability of *V. cholerae* to kill *G. mellonella* larvae and form biofilms in these hosts imitates the pathogenic potential with mammalian model organisms (Nuidate et al., 2016; Bokhari et al., 2017).

*Vibrio cholerae* persists in the aquatic environment between outbreaks where they fight with different predators and environmental stressors. Some models were developed to study the environmental survival of *V. cholerae*. The mannose-sensitive hemagglutinin (MSHA) type IV pilus is crucial for attachment and initiation of colonization of *V. cholerae* in the pharynx of the worm, *C. elegans*, which could be linked to a fitness advantage of *V. cholerae* upon contact with bacterium-grazing nematodes (List et al., 2018). The soft-shelled turtle has potential value as an animal model to study the colonization and the transmission of *V. cholerae* as *V. cholerae* can colonize both on the dorsal side surface and in the intestine of turtles. MSHA is necessary for body surface colonization whereas, toxin-coregulated pili (TCP) or N-acetylglucosamine-binding protein A (GbpA) play important roles for colonization in the intestine (Wang et al., 2017). The type VI secretion system (T6SS) of *V. cholerae* is capable of conferring virulence toward eukaryotic and prokaryotic hosts. VasX, a functional protein for T6SS confers the virulence of *V. cholerae* toward *Dictyostelium discoideum*, a species of soil-dwelling amoeba commonly known as slime mold (MacIntyre et al., 2010; Miyata et al., 2011). These models are also very important to demonstrate the lifestyle of aquatic organisms in the presence of the aquatic bacterium, *V. cholerae*.

Zebrafish (*D. rerio*) are an emerging non-mammalian model to investigate *V. cholerae* pathogenesis, including colonization, transmission, host response, and competition with intestinal microbiota. The zebrafish is a natural host for *V. cholerae* which means *V. cholerae* can colonize the zebrafish intestine without any modification to the host’s microbiota (Runft et al., 2014). *V. cholerae* infection can be initiated through a natural, oral route in zebrafish, which eventually leads to human cholera-like symptoms including liquid stool in excreted water (cloudy water) and secretion of mucin and protein in excreted water within 24 h of initial exposure (Runft et al., 2014; Mitchell et al., 2017; Nag et al., 2018b). Zebrafish also show promise to study innate and adaptive immune responses during *V. cholerae* infection (Farr et al., 2021, 2022). Studies in the zebrafish model have been informative regarding *V. cholerae*’s interaction with gut microbiota and the involvement of T6SS in this interaction (Logan et al., 2018; Breen et al., 2021b). However, as most non-mammalian studies remain to be validated in either human or small mammal cholera models, their applicability to our understanding of human cholera may be limited (Sit et al., 2022).

## Expansion in understanding the interactions between *Vibrio cholerae* and host intestinal microbiota

The human gut microbiome contains the majority of commensal bacteria in the body (Qin et al., 2010; Sender et al., 2016). In humans, the dominant phyla are Firmicutes and Bacteroidetes, though the composition of the gut microbiome can vary among individuals. Intrinsic factors, including host genetics, age, and sex, and extrinsic factors, such as diet and lifestyle, all affect the gut microbiome (Arumugam et al., 2011; Faith et al., 2011; Schmidt et al., 2018). The mucus lining of the gut is also an important component. The mucus layer acts as a natural barrier to protect the intestinal epithelium. It is primarily made of heavily-glycosylated proteins called mucins that are metabolized by the resident bacteria that reside in the intestinal mucus layer. Therefore, the mucus itself must be present to maintain a diverse microbiome (Kashyap et al., 2013; Li et al., 2015; Sicard et al., 2017).

In terms of microbiome models to study *V. cholerae* outside of human fecal samples, experimentation is limited. Mammalian animal models commonly used for *V. cholerae* infection are not ideal for observing microbiome dynamics after colonization. Infant and adult mice, or the adult rabbits used in ileal loop or RITARD models, lack a complex gut microbiome due to age, antibiotic use for colonization, or surgery, respectively (Sawasvirojwong et al., 2013; Matson, 2018). One option is a human or mouse fecal transplant into axenic or gnotobiotic mice to observe *V. cholerae* colonization in the presence of a “humanized” mouse microbiome (You et al., 2019; Alavi et al., 2020). Non-mammalian animal models that are colonized by *V. cholerae* present an advantage due to the fecundity and shorter development period. The *Drosophila* model possesses a simple gut microbiome of low diversity that can be easily manipulated (Wong et al., 2011). The zebrafish model has the advantage of being a natural *V. cholerae* host, and zebrafish are able to keep complex intestinal microbiomes largely intact throughout the entire period of *V. cholerae* exposure, infection, and clearance; the noninvasive method of infection is particularly relevant (Senderovich et al., 2010; Wong et al., 2011; Runft et al., 2014; Breen et al., 2021a). The affordability of high-throughput sequencing of the 16S ribosomal subunit has allowed for more in-depth microbiome studies within the past decade; therefore, the diversity and dynamics of gut microbiomes in relation to pathogens and general perturbation is of growing interest (Gibbons and Gilbert, 2015).

### *Vibrio cholerae* resistance to gut microbiome dynamics

Depending on the biotype or strain, *V. cholerae* can utilize multiple defenses against the gut microbiome. All *V. cholerae* strains have the type six secretion system (T6SS) which, if functional, directly injects toxic effectors into eukaryotic cells and bacteria (Bachmann et al., 2015). The T6SS is one method *V. cholerae* uses to interact with the host and its resident microbiota. The bacterial dynamics can be complex. In the *Drosophila* model, *V. cholerae* with a functional T6SS inhibited host intestinal repair only when three common fly commensals were present altogether, rather than individually (Fast et al., 2020). In infant mice, *V. cholerae* expressing T6SS were able to compete against commensal *Escherichia coli*, with the *E. coli* demonstrating a 300-fold drop in CFU count per small intestine homogenate when compared to the group infected with *V. cholerae* expressing a defective T6SS (Zhao et al., 2018). This suggests that T6SS plays a significant role in colonization of the murine gut. Conversely, T6SS is not necessary for *V. cholerae* colonization of the conventionally-raised adult zebrafish gut. However, when comparing fish infected with T6SS-deleted *V. cholerae* versus wildtype, the amount of other *Vibrio* spp. increased. Perhaps *V. cholerae* uses T6SS as a form of competition to prevent commensal *Vibrio* species from proliferating (Unterweger et al., 2014; Breen et al., 2021b). In gnotobiotic zebrafish larvae, the T6SS of *V. cholerae* O1 El Tor biotype strain promoted an increase in intestinal movement to expel the inoculated commensal, *Aeromonas veronii*. This clearance then allowed for *V. cholerae* colonization (Logan et al., 2018).

*Vibrio cholerae* can interact with specific bacterial species of the microbiome to potentially improve its own colonization. In one study using predictive taxa from an algorithm analyzing fecal samples from Bangladeshi household contacts of cholera patients, certain bacterial species present were selected for *in vitro* study. *V. cholerae* was then exposed to these species and grown in nutrient-poor or nutrient-rich culture. *V. cholerae* growth with *P. aminovorans* in nutrient-rich culture was significantly increased when compared to growth with other species identified from the collected fecal samples (Midani et al., 2018). This data, albeit *in vitro*, could further support the idea that interactions with certain members of the microbiome are advantageous for *V. cholerae*.

*Vibrio cholerae* can also use gut components to compete against other bacteria. One survival study loaded *V. cholerae* with either a mucin or a gelatin control, and compared the recovered bacterial load from each condition. An O1 strain of *V. cholerae*, C6706, normally represses its T6SS in laboratory settings. In the presence of mucin, C6706 activated its T6SS to compete against other strains of *V. cholerae* that maintained an inactivated T6SS. These results did not occur in the gelatin control. This indicates a component of the mucin was necessary to activate a functional T6SS in C6706 (Bachmann et al., 2015).

### Altered gut microbiome composition following *Vibrio cholerae* infection

Once *V. cholerae* reaches the upper small intestine in humans, it comes into contact with the relatively sparse duodenal microbiota. Fecal sample culture studies characterized the microbiome composition during and after diarrheal symptoms. The gut mucosa sloughs off, resulting in the characteristic rice-water stool. The immediate effect is a drastic decrease in diversity, primarily due to the physical efflux of the mucosa containing the resident gut microbes. Stool collected from Bangladeshi adults during the acute phase of diarrheal symptoms showed dominance of *V. cholerae* bacteria and a significant decrease in non-*Vibrio* bacteria (Hsiao et al., 2014; David et al., 2015). Hsiao et al. (2014) also detected 343 bacterial species that colonized the human gut during and after diarrheal symptoms, suggesting some bacteria are able to remain and recolonize following *V. cholerae* infection. Hours after oral rehydration therapy (ORS) was started, *Streptococcus* and *Fusobacterium* species dominated the microbiome. This overall composition was significantly different from the healthy adult microbiome controls. One caveat of human fecal studies is that *V. cholerae* colonizes the upper SI and not the colon, whereas most bacteria harvested in fecal samples are from the colon, so direct effects of *V. cholerae* on the SI microbiome are difficult to assess.

Effects of *V. cholerae* infection on the composition of the intestinal microbiome are also observed in the zebrafish model. Depending on the strain of *V. cholerae* used and whether a functional T6SS was present or absent, the microbiome profile of adult zebrafish was transiently changed following infection (Breen et al., 2021b). Using quantitative PCR (qPCR), some *V. cholerae* strains were also determined to cause an increase in overall bacterial load of the fish intestine.

### Gut microbiome composition can inhibit *Vibrio cholerae* colonization

The gut has a variety of defenses that can work against non-commensals. One important factor in the human intestine is bile, with bile acid being one major component used in digestion to solubilize lipids. Resident gut bacteria can metabolize these bile acids *via* bile salt hydrolases (Hung and Mekalanos, 2005; Song et al., 2019). This bile acid deconjugation can inhibit *V. cholerae* T6SS expression, perhaps due to a carboxylic acid group present in a bile salt, although the mechanism of action is not known (Bachmann et al., 2015). The resident gut microbiome can also produce components against non-commensal bacteria. Two human commensal *Bifidobacterium* species and a commensal *B. subtile* species were able to individually inhibit or decrease *V. cholerae* T6SS-mediated killing (Bachmann et al., 2015).

Another gut commensal, *E. coli*, can produce a genotoxin called colibactin, which is commonly associated with damaging DNA in host epithelial cells (Dougherty and Jobin, 2021). Through transposon mutagenesis, Chen et al. identified three *E. coli* mutants from mouse small intestine that were unable to compete against *V. cholerae*. These mutants all had a disruption of the polyketide synthase island, which encodes colobactin. This carried over to analyzing published shotgun metagenomics sequencing of fecal samples from households with a confirmed cholera case. When compared to asymptomatic or uninfected fecal samples, those collected from symptomatic individuals had significantly lower relative abundance reads of *clb*, a synthase involved in activating colibactin synthesis (Chen et al., 2022). Probiotic *E. coli* strains have also been found to be protective against *V. cholerae* infection using the zebrafish model (Nag et al., 2018a).

*Blautia obeum*, of the core human gut phyla Firmicutes, may also play a role in a *V. cholerae* infection. *B. obeum* produces the DPO autoinducer, which activates a regulatory cascade that may inhibit *V. cholerae* biofilm formation and toxin production (Papenfort et al., 2017). DPO can also inhibit AphA, a transcription factor that regulates virulence gene expression in *V. cholerae* (Herzog et al., 2019). In homogenized suckling mouse intestine colonized with a reporter *V. cholerae* and *B. obeum*, expression of TcpA, the primary structural subunit of the toxin co-regulated pilus (TCP) required for colonization, was significantly reduced. Conversely, in human fecal samples, a higher abundance of *V. cholerae* was associated with a lower amount of *B. obeum* (Alavi et al., 2020).

### Limitations to microbiome and *Vibrio cholerae* studies

The complexity of the human gut microbiome is difficult to standardize, not only in composition but in computation. Variation in bioinformatics analysis can lead to different conclusions. The more common method of clustering 16S rRNA sequences based on 97% identity generates operational taxonomic units (OTUs). This analysis uses the Mothur pipeline as a clustering method. A newer denoising method, DADA2, generates amplicon sequence variants (ASVs) that provide higher sensitivity and variation specificity. These methods used on 16S rRNA gene amplicon datasets can lead to different results in taxonomic assignments, alpha diversity, and beta diversity than the OTU method on the same dataset (Prodan et al., 2020; Straub et al., 2020; Chiarello et al., 2022). Low abundance OTUs and ASVs can also skew the data, which may be relevant during the decreased gut microbiota following mucosal efflux (Prodan et al., 2020). Additionally, the widely used method of 16S rRNA gene sequencing is not reliably species-specific. This can pose a problem in gut microbiome identification, as this microbiome is composed of an estimated 10^13^ microbial cells with thousands of bacterial species (Luckey, 1972; Leviatan et al., 2022).

## Cholera disease presentation and epidemiology

As previously mentioned, *V. cholerae* strains of the O1 serogroup have been subdivided into two biotypes: Classical and El Tor. Though rarely occurring now, Classical infections were characterized by robust action of the CT and severe diarrheal symptoms lasting about 3 days (Hu et al., 2016). Initial infections with the El Tor biotype were mild, often failing to result in any cholera symptoms, but these infections were persistent, with *V. cholerae* isolates found in the stool of infected individuals for 1–2 weeks. The binding subunit of CT encoded by early El Tor strains differs structurally by 2 amino acids compared to the Classical CT which is hypothesized to account for the disparity in disease severity exhibited by Classical and El Tor infections (Raychoudhuri et al., 2009; Baek et al., 2020). Acquisition of the Classical CTXΦ by El Tor in the early 2000s led to increased disease severity comparable to that caused by Classical strains and of prolonged duration as was characteristic of early El Tor strains (Na-Ubol et al., 2011; Hu et al., 2016). Previous exposure to *V. cholerae* causing symptomatic cholera provides protective immunity against subsequent exposure, though cross-protection is not generally achieved for other serotypes or in cases where initial exposure did not produce symptoms (Leung and Matrajt, 2021).

### Global distribution of cholera and at-risk populations

*Vibrio cholerae* O1 outbreaks largely occur in areas with poor sanitation, limited infrastructure, and minimal or no access to safe drinking water (GTFCC, 2017). Nearly all countries with high cholera burdens also suffer below-average access to basic water and sanitation services (GTFCC, 2017; WHO and UNICEF, 2017). Latin America and the Caribbean, Eastern, Southeastern, Western, Central, and Southern Asia, Northern Africa and Sub-Saharan Africa, and Oceania are all regions identified as lacking a basic drinking water service and/or sanitation services as of 2015 (WHO and UNICEF, 2017). Modelling estimates have revealed 69 countries with endemic cholera—defined as having predicted cholera cases in at least 3 years of a 5-year study period—including, but not limited to, Nepal, China, Indonesia, and several countries in Sub-Saharan Africa (Ali et al., 2015). Additionally, India, Ethiopia, Nigeria, Haiti, the Democratic Republic of the Congo, Tanzania, Kenya, and Bangladesh were identified as having >100,000 annual cases in the same study. In 2016, 80% of all reported cases were located in Haiti, the Democratic Republic of the Congo (DRC), Yemen, Somalia, and the United Republic of Tanzania (WHO, 2017). It is important to note that the vast majority of cholera cases are not reported for many reasons, including lack of laboratory diagnostic capabilities or reporting systems, fear of criticism due to a lack of proper infrastructure, and concern for disrupting trade partnerships or tourism initiatives. A review published in 2020 revealed estimated case numbers in the cholera-endemic countries of India, Pakistan, and the Philippines were 5.8-, 6.7-, and 20.8 times greater than the actual reported number of cases, respectively (Ganesan et al., 2020). The use of modeling to predict cholera outbreaks and regions at heightened risk is a truly noteworthy advancement for epidemiological studies and surveillance efforts (Allan et al., 2016; Ali et al., 2017; Camacho et al., 2018). Endemic cholera regions experience cholera “blooms,” or periods during which the number of *V. cholerae* infections increase dramatically in response to seasonal blooming of zooplankton and phytoplankton which feed copepods that support *V. cholerae* abundance in the environment (Epstein, 1993; Constantin de Magny and Colwell, 2009). This series of events often coincides with weather patterns such as monsoon season in the Bay of Bengal region (Lobitz et al., 2000). Several studies have emerged in the last two decades investigating the role of a changing global climate on *V. cholerae* environmental presence, exogenous gene acquisition, and vector-borne dissemination of this pathogen and the connection to global cholera outbreaks (Lipp et al., 2002; Jutla et al., 2010).

In addition to those living in under-resourced regions, displaced populations, and refugee settings have also experienced significant cholera outbreaks in recent years (Shannon et al., 2019). Cholera outbreaks in South Sudan, Yemen, Cameroon, Nigeria, Tanzania, Uganda, Haiti, and Iraq pose a significant threat to these humanitarian aid settings. Both refugee and impoverished populations lack ready access to rehydration therapy and services and therefore are at higher risk for severe disease and death (CDC, 2020). Children under 5 years of age face disproportionate incidence of infection with *V. cholerae* O1 and experience more severe disease (Deen et al., 2008). Healthcare workers or cholera response workers face an increased risk of exposure to *V. cholerae* O1 infection as well, along with leisure travelers who do not follow food or water safety guidelines or practice proper hygiene. Additional risk factors for more severe disease include individuals with blood type O, achlorhydria, or chronic medical conditions (CDC, 2020). Disaster relief operations also need to be aware of the potential for unintentional transmission of cholera to naïve populations and prepare accordingly. Genetic analysis suggests the introduction of O1/O139 *V. cholerae* strains to regions where it is nonendemic is almost exclusively due to human movement (Domman et al., 2017; Weill et al., 2017; Weil et al., 2019).

Cholera in Haiti over the past decade has clearly demonstrated how devastating the introduction of *V. cholerae* to a naïve population can be. *V. cholerae* was introduced to Haiti following an influx of international aid workers who responded to the catastrophic 7.0 magnitude earthquake in 2010 (Chin et al., 2010; Hendriksen et al., 2011; Frerichs et al., 2012; Katz et al., 2013; Orata et al., 2014). Already one of the poorest countries in the western hemisphere, Haiti also suffered from near total loss of infrastructure, access to clean water, and rampant crime in the wake of this natural disaster. Given this was the first introduction of *V. cholerae* to the Haitian people, the death toll rose rapidly to claim the lives of at least 100,000 people (Vega Ocasio et al., 2023). Cholera has since become endemic to this previously unexposed region. After three consecutive years of zero reported cases, however, the country was declared free of cholera by the Haitian Prime Minister, Dr. Ariel Henry in February 2022. Recent prolonged periods of violence and social unrest have disrupted water treatment infrastructure and restricted access of both citizens and aid workers to the resources needed for effective public health prevention measures against cholera. As a result, an outbreak in late September 2022 has already surged to over 20,000 suspected cases of cholera reported in Haiti as of January 3, 2023 (Vega Ocasio et al., 2023). Nearly 80% of patients were hospitalized, and a very high case-fatality rate of 3.0% has been observed. This rapid resurgence of the bacterial pathogen after a prolonged period of near silence suggests environmental reservoirs of *V. cholerae* have been well-established since its initial introduction, and eradication of the disease is unlikely if the underlying risk factors are not resolved.

### Cholera prevention initiatives

Despite over 150 years of academic study, cholera persists as a significant health hazard in over 60 countries. Several prevention and intervention campaigns to eliminate cholera are currently used around the world. In endemic settings, the use of rapid, affordable, and accurate serotyping techniques is critical for identifying pandemic cholera in the field and implementing swift public health response measures. A lateral flow dipstick method, Cas12a-assisted rapid isothermal detection (CARID), was recently developed to identify O1 and O139 serogroups in complex samples using recombinase-aided amplification and CRISPR-Cas (Lu et al., 2022). Of note, the World Health Organization assembled the Global Task Force on Cholera Control (GTFCC) which has established a roadmap to ending cholera by 2030 and is referenced several times in this text. Disease prevention efforts include implementation of improved drinking water sources and sanitation services and instilling proper hygiene habits, often referred to as the WASH (water, sanitation, and hygiene) campaign (GTFCC, 2017). Educating vulnerable populations about daily practices that can be readily implemented to reduce risk for infection remains one of the most effective methods for disease prevention. The other major area of focus for disease prevention is the stockpiling and administration of cholera vaccines to at-risk populations.

Three oral cholera vaccines (OCVs) are currently approved by the WHO: Shanchol^TM^, Euvichol-Plus®, and Dukoral® (WHO, 2019). The former two OCVs can be administered to adults and children over the age of 1 year, while the latter OCV is approved for individuals starting at age 2 years. Despite the heightened risk posed to very young children by cholera, no vaccine has been approved for infants under 1 year of age. A stockpile of Shanchol™ and Euvichol-Plus® OCVs has been established for use in mass vaccination campaigns, but it is insufficient to cover all those needing it. A major drawback to all current OCVs is the duration of protection. At most, these OCVs provide approximately 70% protection for 3 years, and in areas most affected by cholera, access to preventative medical care is limited or not financially feasible (WHO, 2019). Few advancements have been made in the realm of cholera therapeutics. ORS therapy has remained the standard of care for cholera cases for decades despite the proposal of other intervention methods including administration of unsaturated fatty acids, specifically linoleic acid, to prevent CT production by *V. cholerae* O1 or a commensal *E. coli* with glucose probiotic to inhibit colonization (Plecha and Withey, 2015; Withey et al., 2015; Nag et al., 2018a). Expanding beyond ORS has potential to reduce disease severity, shorten duration of the infection, or prevent infection altogether.

Though *V. cholerae* O1 infection continues to threaten the health and safety of impoverished or displaced populations, elimination of cholera remains an achievable goal for public health professionals. Recommendations for achieving this goal include implementation of educational programs for at-risk populations, installation or maintenance of safe drinking water sources and sanitation services, optimization of disease surveillance methods, expansion of cholera therapeutics, and increased accessibility to and development of new cholera vaccines, particularly designed for young children and to afford increased duration of protection.

## Conclusion

From genomics to therapeutic development, *V. cholerae* research continues to make great strides towards a more complete understanding of how this pathogen survives in the aquatic environment, interacts with hosts, and causes disease. Analyses of evolutionary genetics have identified critical events that must occur for *V. cholerae* to transition from an environmental marine microbe to the pandemic strains that plague countries around the world today. Novel potential reservoirs in which *V. cholerae* continues to acquire new genetic material and gain fitness in the environment and the host are being identified in vertebrate fish in addition to the well-established reservoirs of copepods, phytoplankton, and shellfish. Studies focusing on motility, chemotaxis, and biofilm formation provide key insights to how *V. cholerae* survives in the environment and navigates colonization of the human host. Animal models in mice and rabbits offer means to study CT production and virulence gene expression in mammals while the zebrafish model can be used to gain a well-rounded perspective of natural infection including interactions with other members of the normal microbiome. Both the direct action of the T6SS and indirect activity of rapid fluid loss have been shown to disrupt the composition of the normal intestinal microbiota and open a new avenue of exploration regarding interspecies dynamics during *V. cholerae* infection. Increased understanding of microbiome composition that affords resistance against infection with *V. cholerae* offers potential for use of probiotics to reduce risk of infection and limit disease severity. Disease presentation has shifted with the changing genetic composition of El Tor variants, which exhibit various antimicrobial resistance patterns and a range of symptom severity depending on the exact etiological agent. Advances in cholera disease modeling have enabled better prediction of outbreak scenarios and, in turn, faster implementation of prevention initiatives and response efforts. Taken together, these advancements offer a holistic approach to the study of a persistent pathogen that has plagued the world for centuries. Continued efforts to explore *V. cholerae* dynamics as a model for genomic evolution, bacterial pathogenesis, and its role as a natural member of the aquatic ecosystem are needed, both to advance knowledge in the basic sciences and to develop relevant, effective public health measures to protect the most vulnerable populations.

## Author contributions

MW led the writing of the manuscript. DN and IC contributed to writing the manuscript. JW contributed to the writing, edited the manuscript, and provided funding (grant numbers R01AI127390 and R21AI171072). All authors contributed to the article and approved the submitted version.

## Conflict of interest

The authors declare that the research was conducted in the absence of any commercial or financial relationships that could be construed as a potential conflict of interest.

## Publisher’s note

All claims expressed in this article are solely those of the authors and do not necessarily represent those of their affiliated organizations, or those of the publisher, the editors and the reviewers. Any product that may be evaluated in this article, or claim that may be made by its manufacturer, is not guaranteed or endorsed by the publisher.
